# Pigs: A stepwise RGB-D novel pig carcass cutting dataset

**DOI:** 10.1016/j.dib.2022.107945

**Published:** 2022-02-15

**Authors:** Ian de Medeiros Esper, Luiz Eduardo Cordova-Lopez, Dmytro Romanov, Ole Alvseike, Pål Johan From, Alex Mason

**Affiliations:** a Norwegian Univiersity of Life Sciences - Faculty of Science and Technology, Universitetstunet 3, 1430 Ås, Norway; b Animalia AS, Oslo 0585, Norway

**Keywords:** Pig/pork carcass, Stepwise pig cutting, RGB-D, Pig cutting images, Pig cutting depth data, CHU, Carcass Handling Unit

## Abstract

This paper presents a pig carcass cutting dataset, captured from a bespoke frame structure with 6 Intel® RealSense™ Depth Camera D415 cameras attached, and later recorded from a single camera attached to a robotic arm cycling through the positions previously defined by the frame structure. The data is composed of bags files recorded from the Intel’s SDK, which includes RGB-D data and camera intrinsic parameters for each sensor. In addition, ten JSON files with the transformation matrix for each camera in relation to the left/front camera in the structure are provided, five JSON files for the data recorded with the bespoke frame and five JSON files for the data captured with the robotic arm.

## Specifications Table

**Table:** 

Subject	Animal Science/Food Science/Computer Science
Specific subject area	Food technology, image processing, artificial intelligence, computer vision, pattern recognition, and animal physiology
Type of data	Bag file version 2.0 containing: RGB image, depth data, and camera intrinsic matrix
	JSON file with the transformation matrix in relation to the left/front camera.
How data were acquired	6 Intel® RealSense™ Depth Camera D415 cameras in an inward 360 degrees setup (2 on the right, 2 on top, and 2 on the left).
Data format	Raw
Parameters for data collection	The data were collected in a laboratory, with the same lighting conditions and the first 50 frames were discarded due to brightness adjustment of the camera.
Description of data collection	The data of 25 pigs were saved using an in house software that used Intel®’s Realsense™S DK to save the bag (ver. 2.0) files.
Data source location	Institution: Norwegian Univiersity of Life Sciences
	City/Town/Region: Ås, Viken
	Country: Norway
Data accessibility	Repository name: DataverseNO
	Data identification number: 10.18710/GDGHZR
	URL: https://dataverse.no/dataset.xhtml?persistentId=doi:10.18710/GDGHZR

## Value of the Data

• This dataset provides a step-by-step cut to remove shoulder, ham and split ribs of 25 pigs laid horizontally.
• It provides RGB, depth, intrinsic and extrinsic parameters that researchers can use to investigate in both 2D and 3D environments.
• The data can be used for computer vision and machine learning to improve object recognition, object classification and semantic segmentation of pigs, pig’s parts, and segmentation between skin, muscle, and fat.
• Collection of such data demands skillful resources and specialist equipment, therefore making it costly to collect.
• It can be used to research, develop, and improve robotic and/or intelligent systems for abattoirs and meat processing plants.

## 1 Data Description

Pork is the most consumed meat in the world today [1], but slaughter and process lines are still largely manual [2]. The dexterity required to cut the meat, follow bones and sever ligaments and tendons are not easily replicated by robotic arms [3], [4], [5], [6], [7]. Aiming to develop intelligent systems for the meat industry, a set of data containing the RGB and depth images was captured as part of the research projects *“MeaTable - Robotised cells to obtain efficient meat production for the Norwegian meat industry”*^1^ [8] and *”RoButcher - A Robust, Flexible and Scalable Cognitive Robotics Platform”*^2^.

The bag^3^ container file from ROS [9] was used to store the data. The most important information stored in the file stores are the following:

• *Info*: Camera high-level information, e.g., firmware version, serial number, camera name.
• *Depth Data*: Depth data for every captured frame.
• *Depth Camera Info*: Camera information for the depth frame.
• *Color Data*: Color sensor data for every captured frame.
• *Color Camera Info*: Camera information for the color sensor.

The file contains other information such as exposure, processor temperature, IR emitter, etc. To fully inspect the file the program *rs-rosbag-inspector*, part of the Intel® RealSense™2.0 SDK, can be used.

Ten additional JSON files are provided with the transformation matrix of the cameras. Five transformations for the data captured with the frame and five for the process done by the robotic arm. The transformations are for all the cameras/positions except for the left/front camera/position, as this reference the origin for the transformations.

The dataset is stored in bag files and the naming convention for the files was changed between captures of 2020 and 2021 due to evolution of the methods, as described in the next section.

The naming convention for 2020’s files are the following:

<YYYY−MM−DD>_pig_<number>−<Processed_Part>−Capture<Position>−<Camera_Serial_Number>_<sequence>.bag

• The position can be Low or High
• The process parts are: first (whole carcass), left ham, left ribs, left shoulder, right ham, right ribs, and right shoulder.
• E.g, 2020-06-25_pig_1-LeftShoulder-CaptureHigh-839112060842_1.bag

The naming convention for 2021’s files are the following:

<YYYY−MM−DD>_pig_<number>−<Processed_Part>−Cam_<position>_<sequence>.bag

• Position are from 0 to 5
• E.g, 2021-05-25_pig_2-RightShoulder-Cam_5_3.bag

The relationship between bag files and the transformations are as follows:

Data from 2020 uses Transformations_Frame’s files to reconstruct the 3D from all the cameras. Data from 2021 uses Transformations_Robot’s files to reconstruct the 3D from all the cameras.

**Fig. 1:**
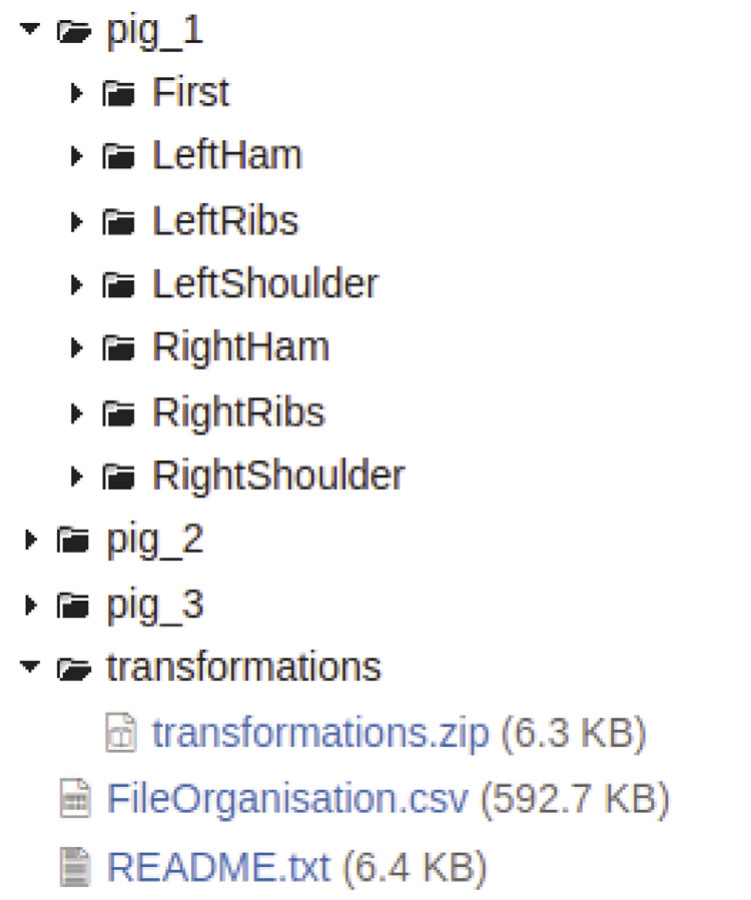
Folder structure in the data repository.

Due to limitations in the data repository, the files were compressed and organized according to the pig number and the part that was being processed during the data capture, as shown in Fig. 1. A complete list with all the files, the pig number, and folder structure is supplied in a CSV file called FileOrganisation.csv, making it easier to create scripts or programs to read a specific set of files.

The process follows the cutting pattern described by [10] and the parts are: first (whole carcass), left ham, left ribs, left shoulder, right ham, right ribs, and right shoulder.

**Fig. 2:**
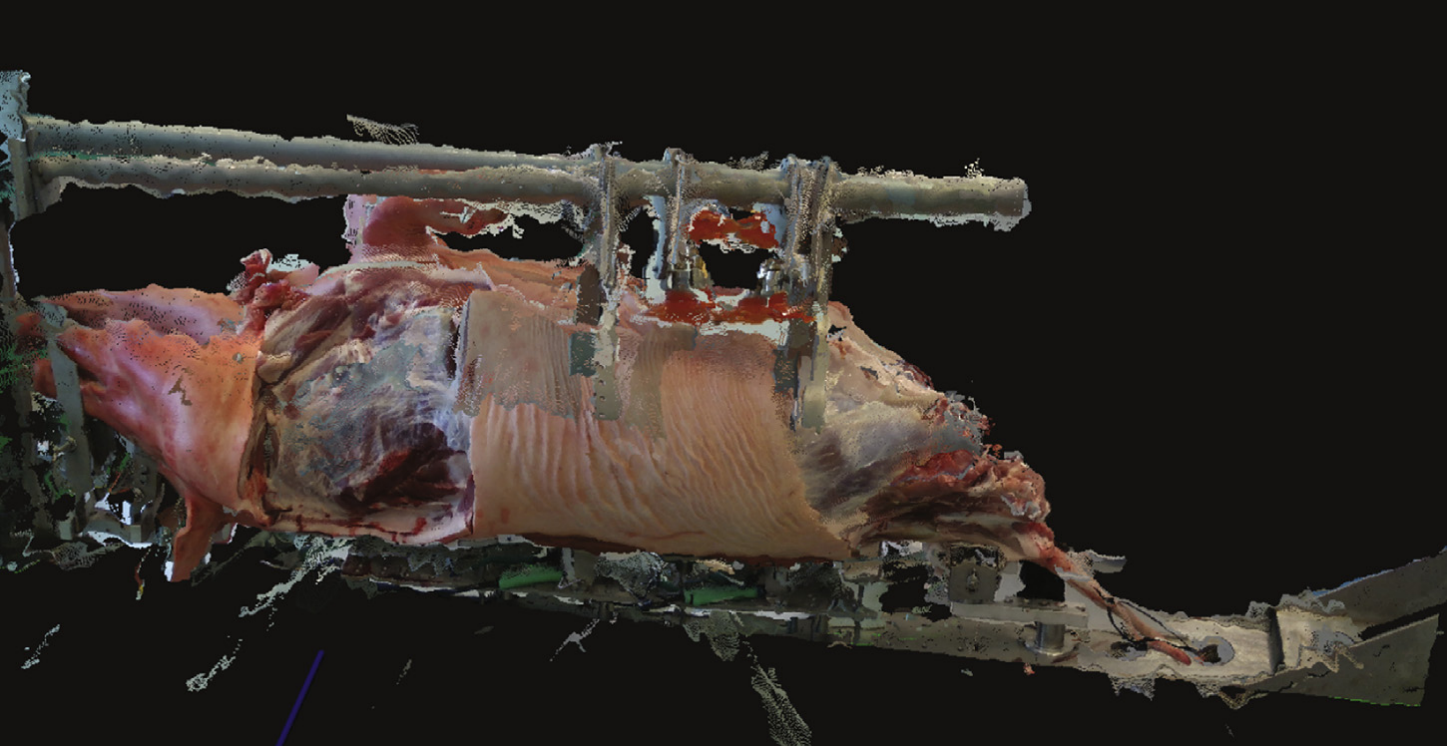
3D point cloud reconstruction using the dataset.

**Fig. 3:**
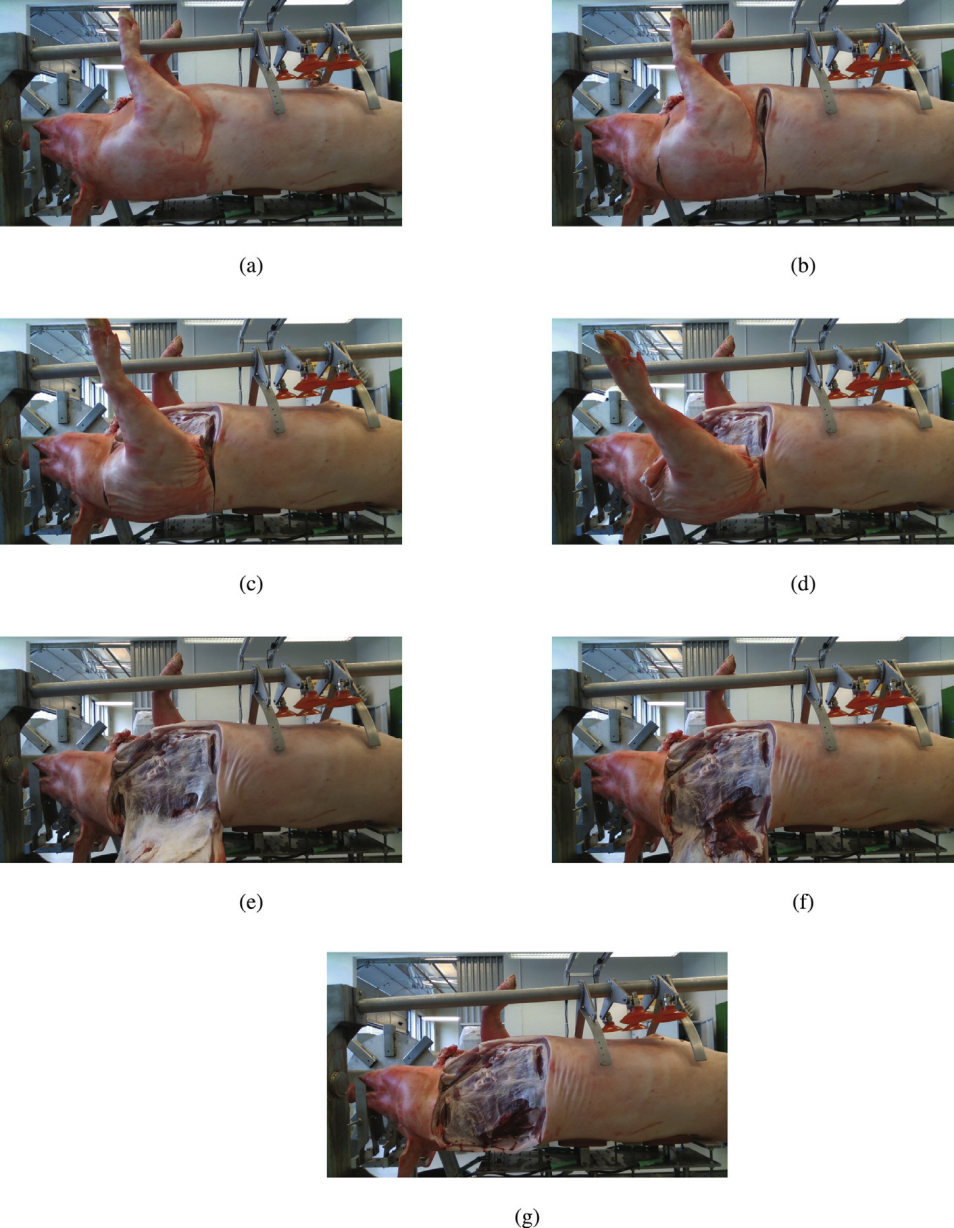
Steps of the cutting of the left shoulder captured by the left/front camera from (a) to (g).

Fig. 2 shows the point cloud of the pig carcass after the removal of the hams and one shoulder, reconstructed using the transformations in the JSON files. Fig. 3 shows the color frame of the steps of cutting the left shoulder as captured by one camera.

## 2 Experimental Design, Materials and Methods

**Fig. 4:**
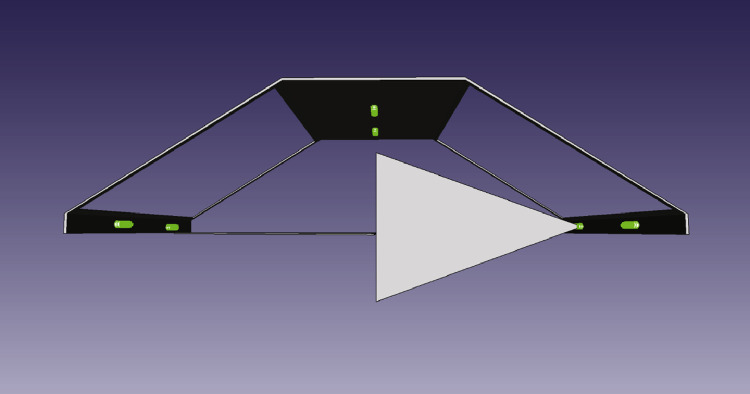
3D Cad of the camera support with cameras in green.

**Fig. 5:**
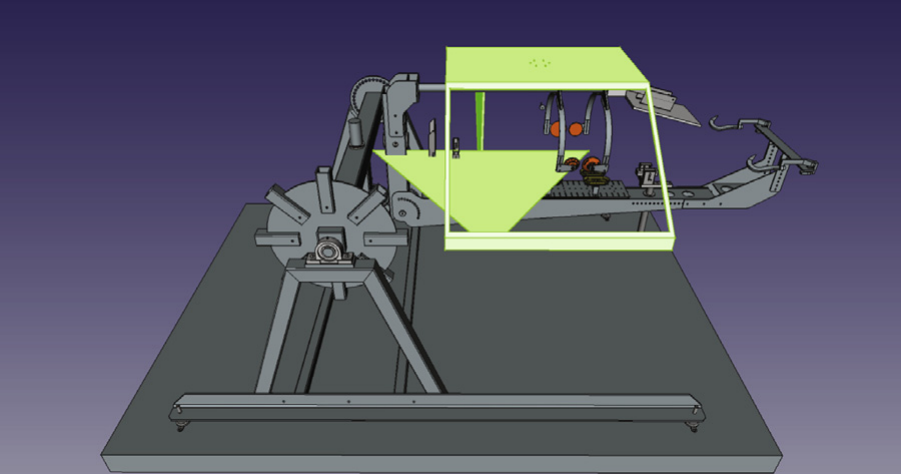
CHU with the capture frame in green.

**Fig. 6:**
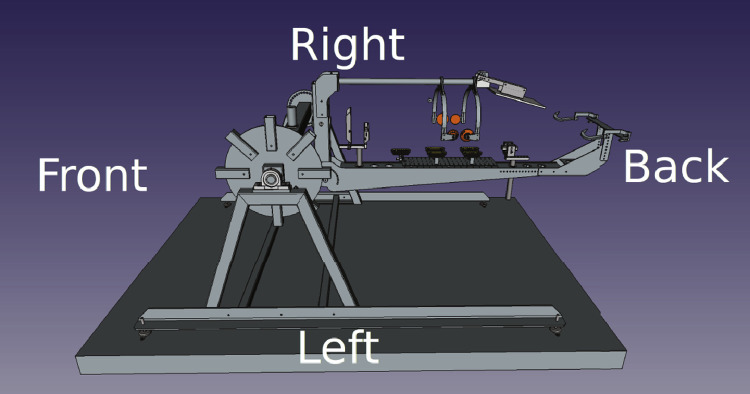
CHU with the sides.

**Fig. 7:**
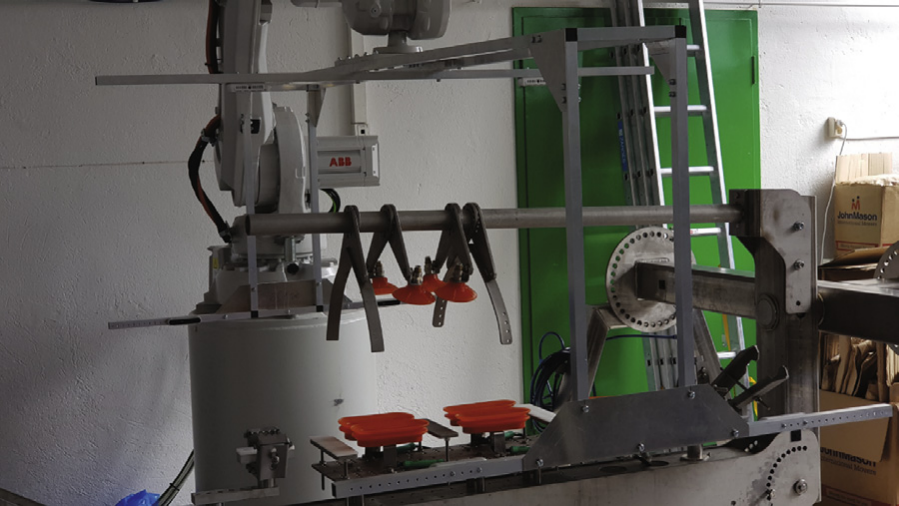
Camera Support and CHU.

Two different methods were used to capture the data. The first data, captured in 2020, used a bespoke frame as seen in Fig. 4. The frame surrounds a customized machine called the carcass handling unit (CHU) as shown in Fig. 5 where the camera frame is shown in green. The cameras are referenced as left/right/up and front/back. The left/right is defined in relation to the CHU, not in relation to the pig as it can rotate once grabbed by the CHU. The support frame has its left, right, front, and back sides defined as seen in Fig. 6. The physical frame structure can be seen in Fig. 7.

The cameras chosen were the Intel® RealSense™ Depth Camera D415 [11] and they are positioned according to the serial number as follows:

• Left/Front: 839112061131
• Left/Back: 839112061608
• Up/Back: 839112061317
• Up/Front: 839112061134
• Right/Front: 907222062247
• Right/Back: 839112060842

**Fig. 8:**
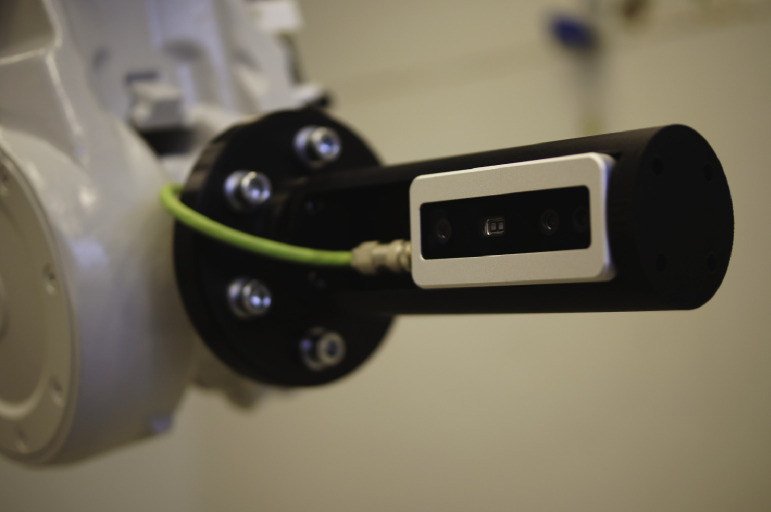
Camera attachment to the robotic arm.

In 2021 as an evolution of the capturing system, a single camera attached to a robot was used as seen in Fig. 8. The advantages of using a robot are the repeatability^4^ of the robot. In this case, is 0.06mm according to ABB’s document [13], its speed, and the possibility of using only one camera. The robot cycles to almost the same positions as the camera frame used previously, thus the data is very similar.

Since only one camera is being used, the file name now uses an integer from 0 to 5, representing the position where the data were recorded, as shown below:

• Left/Front: Cam_0
• Left/Back: Cam_1
• Up/Back: Cam_2
• Up/Front: Cam_3
• Right/Front: Cam_4
• Right/Back: Cam_5

The files were recorded using the Intel® RealSense™ SDK 2.0, using a bespoke software application. When using the frame structure, the first 60 frames of every capture were discarded due to exposure correction variations, then 30 frames of data were recorded. Using the robot, the camera can be continuously turned on, not making the initial exposure variation an issue, thus there is no need to discard frames.

The left side of the pig was captured in more detail following a cut-by-cut capture process, while the right side was imaged prior to cutting, after the initial surface cuts, at the midway cutting point, just prior to removal, and after the final cut (i.e. limb removed). This was necessary due to time constraints imposed by the carcass’s internal organs remaining intact.

## Ethics Statement

The data captured in this work is from cadavers. Entire carcasses (Norwegian Landrace, male and female) were purchased from an abattoir authorised to undertake slaughter (Fatland, Oslo, Norway) according to the Norwegian Food Safety Authority, Mattilsynet. The carcasses were received post stunning, bleeding and washing, but prior to butchering. Following guidance from Mattilsynet, the cadavers used in this work did not re-enter the food supply chain in any form, and were disposed of via a third party authorised to destroy animal waste. The authors therefore applied the 3R principle: Reduction (using as few cadavers as possible to achieve necessary goals), Replacement (using models or other methods to test systems prior to the use of cadavers) and Refinement (use of the cadavers for multiple studies prior to disposal, including collaborative work with other projects where possible). By publishing this data set, the authors hope to further aid reduction of cadaver use in the development of robotic or automation systems for meat processing.

## CRediT authorship contribution statement

**Ian de Medeiros Esper:** Methodology, Data curation, Software, Writing – original draft. **Luiz Eduardo Cordova-Lopez:** Methodology, Resources. **Dmytro Romanov:** Resources. **Ole Alvseike:** Conceptualization, Supervision, Resources. **Pål Johan From:** Supervision. **Alex Mason:** Conceptualization, Methodology, Supervision, Writing – review & editing.

## Declaration of Competing Interest

The authors declare that they have no known competing financial interests or personal relationships which have, or could be perceived to have, influenced the work reported in this article.

## Footnotes

1 https://www.nmbu.no/en/projects/node/34494.

2 https://robutcher.eu/.

3 http://wiki.ros.org/Bags.

4 *“Repeatability is the ability of the robot to return its TCP (tool center point) to the same position, repeatedly from the same direction.”*[12].
